# Exploring the roles of oxygen species in H_2_ oxidation at β-MnO_2_ surfaces using operando DRIFTS-MS

**DOI:** 10.1038/s42004-022-00717-0

**Published:** 2022-08-20

**Authors:** Jiacheng Xu, Tiantian Zhang, Shiyu Fang, Jing Li, Zuliang Wu, Wei Wang, Jiali Zhu, Erhao Gao, Shuiliang Yao

**Affiliations:** 1 School of Environmental and Safety Engineering, Changzhou University, Changzhou, China; 2 School of Material Science and Engineering, Changzhou University, Changzhou, China; 3 Advanced Plasma Catalysis Engineering Laboratory for China Petrochemical Industry, Changzhou, China

**Keywords:** Catalytic mechanisms, Heterogeneous catalysis, Materials for energy and catalysis, Infrared spectroscopy, Mass spectrometry

## Abstract

Understanding of the roles of oxygen species at reducible metal oxide surfaces under real oxidation conditions is important to improve the performance of these catalysts. The present study addresses this issue by applying a combination of operando diffuse reflectance infrared Fourier transform spectroscopy with a temperature-programmed reaction cell and mass spectrometry to explore the behaviors of oxygen species during H_2_ oxidation in a temperature range of 25–400 °C at β-MnO_2_ surfaces. It is revealed that O_2_ is dissociated simultaneously into terminal-type oxygen (M^2+^-O^2–^) and bridge-type oxygen (M^+^-O^2–^-M^+^) via adsorption at the Mn cation with an oxygen vacancy. O_2_ adsorption is inhibited if the Mn cation is covered with terminal-adsorbed species (O, OH, or H_2_O). In a temperature range of 110–150 °C, OH at Mn cation becomes reactive and its reaction product (H_2_O) can desorb from the Mn cation, resulting in the formation of bare Mn cation for O_2_ adsorption and dissociation. At a temperature above 150 °C, OH is reactive enough to leave bare Mn cation for O_2_ adsorption and dissociation. These results suggest that bare metal cations with oxygen vacancies are important to improve the performance of reducible metal oxide catalysts.

## Abstract (web summary)

Understanding the roles of oxygen species at reducible metal oxide surfaces is key to improving catalyst performance. Here, a combination of operando diffuse reflectance infrared Fourier transform spectroscopy and mass spectrometry are employed to explore the behaviours of oxygen species during H_2_ oxidation at β-MnO_2_ surfaces.

## Introduction

Oxygen vacancy (OV) defects at reducible metal oxide surfaces play a key role in a heterogenous catalytic oxidation process^1–4^. In 1954, Mars and van Krevelen reported that the oxidation of organic compounds on V_2_O_5_ includes V_2_O_5_ reduction by an organic compound and the subsequent oxidation of V_2_O_5_ by O_2_^5^. This reduction and oxidation mechanism had been verified the OVs at the atomic level for the oxidation of CO on RuO_2_ (110) surfaces using scanning tunneling microscopy (STM) in conjunction with density-functional theory (DFT) calculations^6^. This has induced a boost in studies to identify OVs on metal oxide surfaces. For example, OVs have been identified on the surfaces of rutile TiO_2_ using high-resolution STM^7^. Other studies have demonstrated that many types of OVs with different catalytic reaction characteristics can exist on metal oxide surfaces. OVs have been observed on metal oxide surfaces in association with three metal (M) and oxygen (O) groups (M=O, M–O–M, and M_3_–O)^8^. Moreover, the local structures of OVs on the treated and untreated surfaces of CeO_2_ (110) crystal planes have been elucidated using STM in conjunction with DFT calculations^9^.

Recent reviews have summarized the methods that can be applied to characterize oxygen species at catalyst surfaces^10^ (Supplementary Table S1). An overview has focused on understanding the roles of OVs playing in the oxidation reaction at reducible metal oxide surfaces^11^. For example, the dissociation of O_2_ at OVs was found to greatly impact oxygen adsorption on TiO_2_ (110) surfaces, where one O atom from the dissociated O_2_ molecule is postulated to fill an OV and the second O atom deposited at the five-coordinate Ti^4+^ site^12^. The roles of oxygen atoms and molecules at catalyst surfaces and the properties of OVs have also been the subject of a recent review^13^.

The importance of OVs has led to the development of numerous strategies for increasing the concentration of OVs in metal oxide catalysts. Some success has been achieved via doping with secondary metal ions and nano structuring^14,15^, and the doping strategy has been expanded to develop four-layer metal oxide catalysts (CuO/VO_x_/Ti_0.5_Sn_0.5_O_2_) with layers composed of synergistic OV concentrations^16^. The dispersal of metal ions on the surfaces of metal oxides has also been demonstrated to increase the concentration of OVs effectively^17,18^. These strategies have been widely used in photocatalytic materials, electrocatalytic materials, thermal catalytic materials, and optical materials^19–21^. However, effective methods to improve the performance of metal oxide catalysts are influenced by current characterization technologies. Therefore, it is required to find an effective characterization technology to identify OVs and understand oxidation mechanisms that occur at the surfaces of metal oxides under real reaction conditions.

The operando diffuse reflectance infrared Fourier transform spectroscopy (DRIFTS) is a powerful technology that can identify surface species on a catalyst under real reaction conditions. Ye et al. found that toluene adsorption and reaction with OVs can effectively reduce the accumulation of by-products^22^. Li et al. investigated the structure-performance relationships of α, β, γ, and δ-MnO_2_ catalysts, they found that toluene adsorption is promoted by rapid dehydrogenation of methyl groups on the surface of *δ*-MnO_2_^23^. Yao et al. used the combination of DRIFTS with a mass spectrometry (MS) to observe the functional groups on the catalyst surface and the changes in MS signals of gaseous components during the catalytic oxidation of toluene on CeO_2_^24^.

Due to its multiple valence states and structural diversity (e.g., tunneling (α, β, and γ-MnO_2_) and layered (δ-MnO_2_) structures), MnO_2_ is an important functional metal oxide material^25–27^. β-MnO_2_ has a thermodynamically stable phase and high crystallinity, and become one of the hot spots in current researches^28,29^.

The present work addresses these issues by combining an operando DRIFTS with a temperature-programmed reaction (TPR) cell and MS to explore the behaviors of OVs and adsorbed oxygen species at β-MnO_2_ surfaces during H_2_ oxidation reaction conducted in the temperature range of 25–400 °C. The roles of OVs in H_2_ oxidation process are explored according to relations between OVs and oxygen species, which in turn reveal interactions between surface oxygen species with H_2_ at different reaction temperatures.

## Results and discussion

### Catalyst characterization

**Fig. 1:**
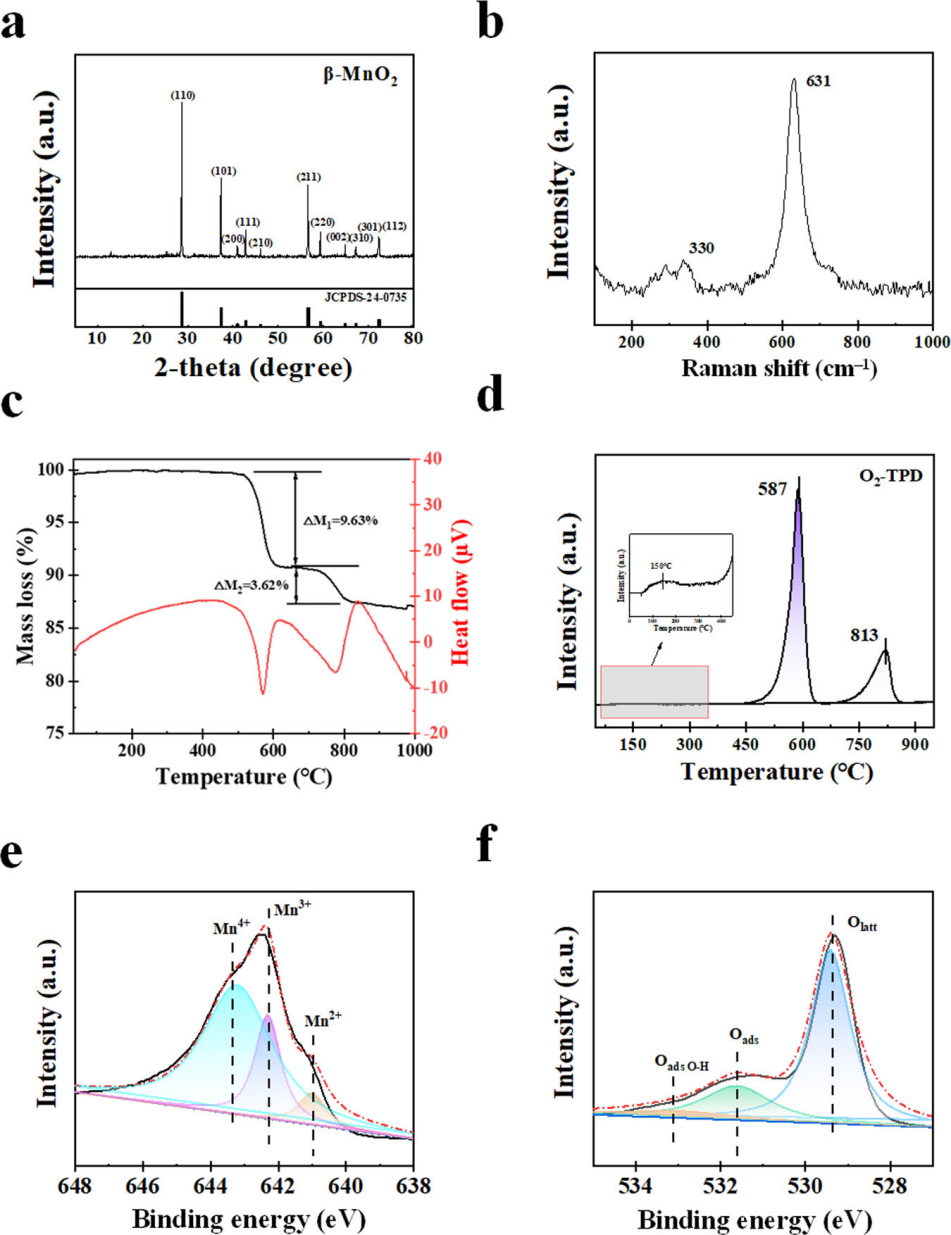
Catalyst characterization of β-MnO_2_. **a** XRD patterns. **b** Raman spectrum. **c** TG profile. **d** O_2_-TPD profile. **e** Mn2p XPS spectrum. **f** O1s XPS spectrum.

The crystal structure of β-MnO_2_ was confirmed using X-ray diffraction (XRD). β-MnO_2_ has good crystallization and no obvious crystal defects (Fig. 1a). High-resolution transmission electron microscope (HRTEM) image of β-MnO_2_ is shown in Supplementary Fig. S1. The well-identified periodic lattice fringes of 2.41 and 3.15 nm are corresponding to the interplanar distances of (101) and (110) facets of β-MnO_2_. Whereas severe blurring of the lattice fringes is also found (highlighted by red rectangles), suggesting the existence of OVs at β-MnO_2_ surfaces^30^.

Figure 1b shows the Raman scattering spectrum of β-MnO_2_. The band at 630 cm^**–**1^ corresponds to the tensile pattern of the [MnO_6_] octahedron, and the band at 330 cm^**–**1^ is assigned to the metal-oxygen chain of Mn–O–Mn in the MnO_2_ octahedral lattice, indicating the presence of a well-developed rutile-shaped skeleton^31^.

Thermogravimetric (TG) analysis result shows that the weight loss of β-MnO_2_ is not obvious below 500 °C (Fig. 1c). This is due to the coordination of Mn and O in the phase structure is close to saturation, and the phase tunnel structure is stable. The weight loss at higher temperatures is attributed to the removal of lattice oxygen, resulting in the reduction of MnO_2_ to Mn_2_O_3_ (between 500 and 600 °C) with a weight loss of 9.63% and to Mn_3_O_4_ (between 720 and 820 °C) with a weight loss of 3.62%^32^.

O_2_ temperature-programmed desorption (O_2_-TPD) was used to observe the O_2_ desorption from β-MnO_2_ (Fig. 1d). There is a small desorption peak around 150 °C, which is a signal of surface oxygen desorption. When the temperature reaches about 587 and 813 °C, two obvious desorption peaks appear. The small peak at around 150 °C is due to O_2_ desorbed from β-MnO_2_, the peaks at 587 and 813 °C are related to the desorption of lattice oxygen and bulk lattice oxygen^33,34^.

X-ray photoelectron spectroscopy (XPS) was used to measure the valence states of Mn and the types of O at β-MnO_2_ surfaces (Fig. 1e, f and Table 1). The fraction ratios of Mn^3+^ and Mn^4+^ are 32.0% and 68.0%, respectively, indicating that β-MnO_2_ is oxidizable and reducible. O_1s_ spectrum can be divided into lattice oxygen (O_latt_) at 529.2 eV and adsorbed oxygen/surface hydroxyl groups (O_ads_ and (OH)_ads_) at 531.7 and 533.2 eV (Fig. 1f)^35,36^. The fraction ratio of O_latt_ is 77.2%, indicating the presence of OVs at β-MnO_2_ surface.

**Table 1:** Mn2p, O1s binding energies, and the corresponding parameters.

Elements	Assignment	Peak position (eV)	Fraction (%)
Mn2p	Mn^3+^	642.3	32.0
	Mn^4+^	643.4	68.0
O1s	O_latt_	529.2	77.2
	O_ads_	531.7	16.2
	(OH)_ads_	533.2	6.6

### H_2_ oxidation by surface oxygen species in the absence of O_2_

**Fig. 2:**
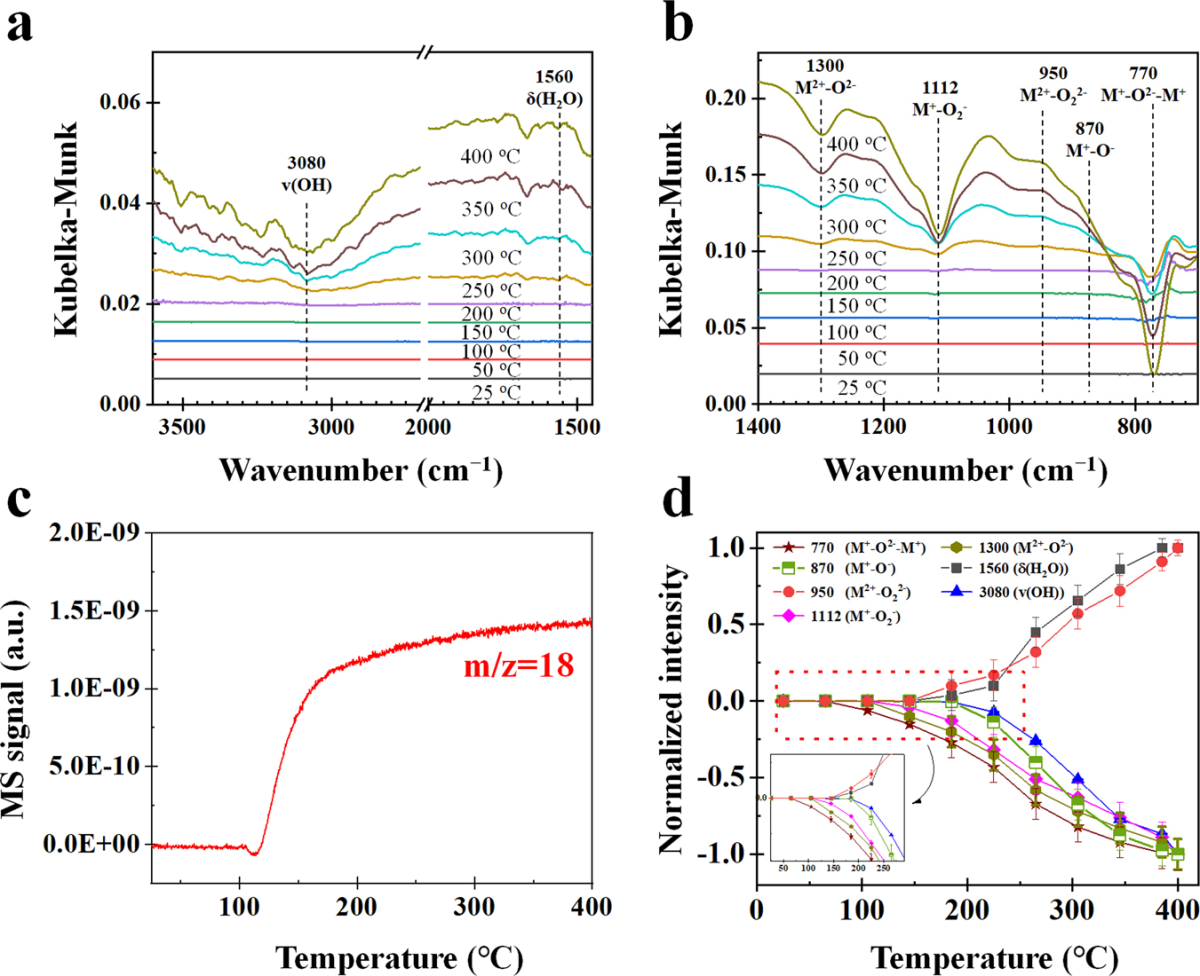
Experimental results of H_2_ oxidation by oxygen species at β-MnO_2_ surfaces in H_2_/Ar as a function of temperature. **a**, **b** DRIFTS spectra. **c** MS signal. **d** Normalized intensities where the error bars are the standard deviations obtained by measuring the peak heights more than three times.

The DRIFTS spectra, MS signals, and normalized peak intensities during H_2_ oxidation by oxygen species at β-MnO_2_ surfaces in the absence of O_2_ are shown in Fig. 2. Seven kinds of oxygen species at β-MnO_2_ surfaces can be found, those are bridge-type (M^+^–O^2–^–M^+^) group (750–800 cm^–1^)^37^, terminal-type (M^2+^–O^2–^) group (1300–1400 cm^–1^)^38–40^ (Supplementary Figs. S2–S4 also prove that 1300 cm^–1^ belongs to M=O at β-MnO_2_ surfaces), M^+^–O^**–**^ group (870 cm^–1^)^41^, adsorbed molecular O_2_ groups including M^+^–O_2_^−^ group (1110–1120 cm^–1^)^42,43^ and M^2+^–O_2_^2−^ group (930–960 cm^–1^)^44^, and oxidation products including *δ*(H_2_O) (1520, 1610, and 1640 cm^–1^) and *v*(OH) (3080, 3230, 3530, and 3720 cm^−1^)^45,46^ (Supplementary Table S3).

At a temperature higher than 110 °C, H_2_O MS signal increases obviously (Fig. 2c) and the normalized intensity of M^+^–O^2–^–M^+^ (M–O–M) decreases (Fig. 2d), but the normalized intensity of other surface oxygen species do not change significantly below 110 °C. This finding implies that the bridge-type of oxygen atom in M^+^–O^2–^–M^+^ (M–O–M) first reacts with H_2_ to form gaseous H_2_O and bridge-type OV (M–□–M, where, OV is represented by an empty square □) (Eq. (1)). When the temperature exceeds 150 °C, except M^+^–O^2**–**^–M^+^, the normalized intensities of M^+^–O_2_^−^, M^2+^–O^2**–**^, M^+^–O^**–**^, and *v*(OH) decrease, but the normalized intensities of *δ*(H_2_O) and M^2+^–O_2_^2−^ increase with increasing temperature. These results indicate that M^+^–O^**–**^ (M–O) and M^2+^–O^2**–**^ (M=O) can react with H_2_ above 150 °C to generate H_2_O and terminal-type OV (bare M) (Eqs. (2) and (3)), which leads to an increase in the normalized intensity of *δ*(H_2_O)^47^. M^2+^–O^2**–**^ (M=O) reacts with surface H_2_O to form OH (Eq. (4)), which leads to a decrease in the normalized intensity of *v*(OH) in H_2_O at β-MnO_2_ surfaces.

1 $${{{{{\rm{M}}}}}}{-}{{{{{\rm{O}}}}}}{-}{{{{{\rm{M}}}}}}+{{{{{{\rm{H}}}}}}}_{2}\to {{{{{\rm{M}}}}}}{-}{{\square }}{-}{{{{{\rm{M}}}}}}+{{{{{{\rm{H}}}}}}}_{2}{{{{{\rm{O}}}}}}$$

2 $${{{{{\rm{M}}}}}}{-}{{{{{\rm{O}}}}}}+{{{{{{\rm{H}}}}}}}_{2}\to {{{{{\rm{M}}}}}}+{{{{{{\rm{H}}}}}}}_{2}{{{{{\rm{O}}}}}}$$

3 $${{{{{\rm{M}}}}}}{=}{{{{{\rm{O}}}}}}+{{{{{{\rm{H}}}}}}}_{2}\to {{{{{\rm{M}}}}}}+{{{{{{\rm{H}}}}}}}_{2}{{{{{\rm{O}}}}}}$$

4 $${{{{{\rm{M}}}}}}{=}{{{{{\rm{O}}}}}}+{{{{{\rm{M}}}}}}{-}{{{{{{\rm{H}}}}}}}_{2}{{{{{\rm{O}}}}}}\to 2{{{{{\rm{M}}}}}}{-}{{{{{\rm{OH}}}}}}$$

It is interesting that the normalized intensity of M^2+^–O_2_^2−^ increases with increasing temperature even in the absence of O_2_ (Fig. 2d). The relation of normalized intensities of M^2+^–O_2_^2−^ and M^2+^–O_2_^−^ is correlated (Supplementary Fig. S5). It was found that a standard deviation (*R*^2^) of the relation is 0.965, which clearly indicates that the normalized intensity of M^2+^–O_2_^2−^ is strongly correlated with that of M^+^–O_2_^−^. Li et al. also reported similar phenomena^48^. The conversion reaction between M^2+^–O_2_^−^ and M^2+^–O_2_^2−^ is shown in Eq. (5), where the valence state of the M cation in M^+^–O_2_^−^ is kept constant via M^+^–O_2_^−^ conversion to M^2+^–O_2_^2−^ after the formation of M–□–M.

5 $$\left({{{{{{\rm{O}}}}}}}_{2}^{-}\right){{{{{\rm{M}}}}}}{-}{{{{{\rm{O}}}}}}-{{{{{\rm{M}}}}}}+{{{{{{\rm{H}}}}}}}_{2}\to \left({{{{{{\rm{O}}}}}}}_{2}^{2-}\right){{{{{\rm{M}}}}}}{-}{{\square }}{-}{{{{{\rm{M}}}}}}+{{{{{{\rm{H}}}}}}}_{2}{{{{{\rm{O}}}}}}$$

### H_2_ oxidation by surface oxygen species in the presence of O_2_

**Fig. 3:**
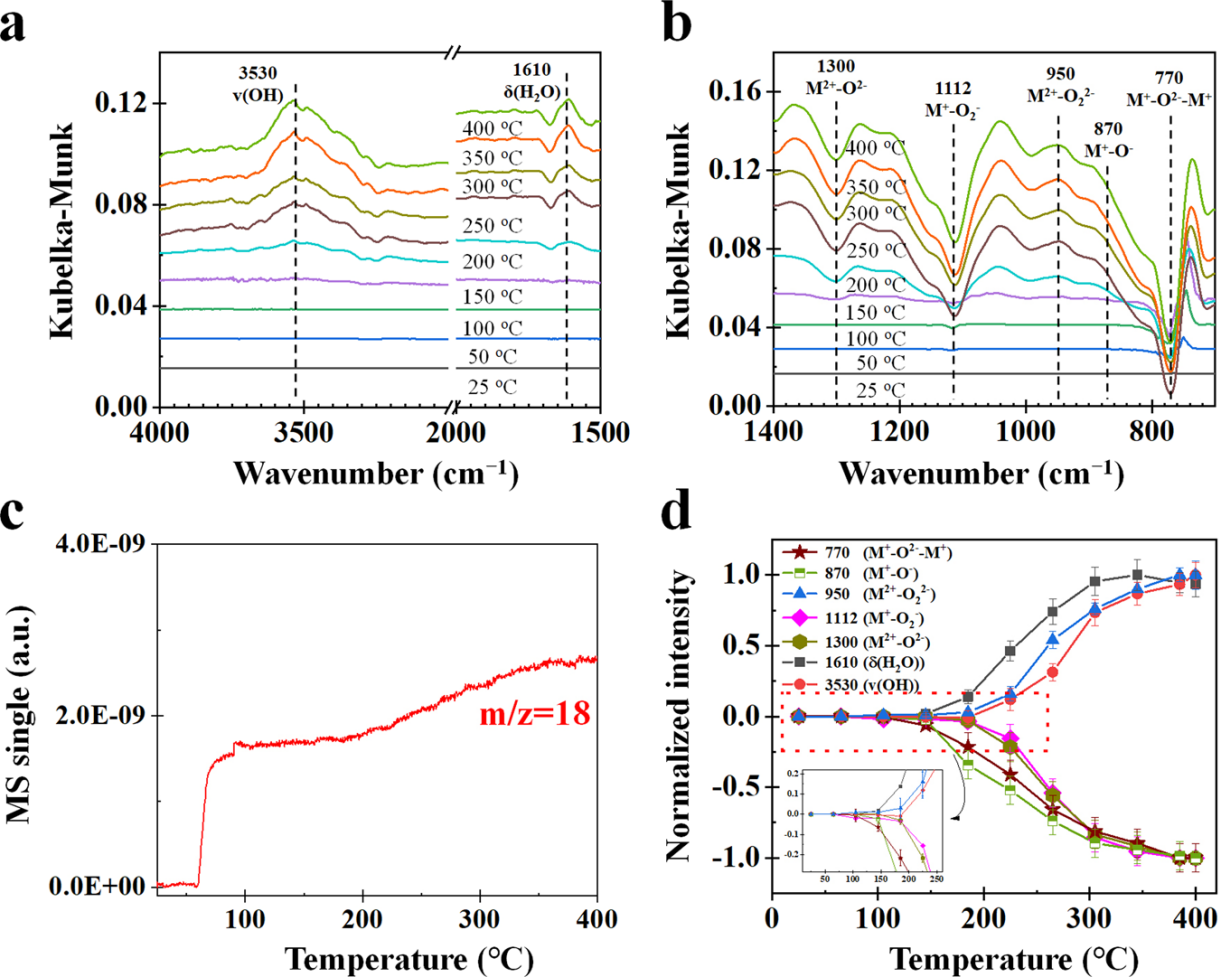
Experimental results of H_2_ oxidation by oxygen species at β-MnO_2_ surfaces in (H_2_ + O_2_) as a function of temperature. **a**, **b** DRIFTS spectra. **c** MS signal. **d** Normalized intensities where the error bars are the standard deviations obtained by measuring the peak heights more than three times.

The DRIFTS spectra, MS signals, and normalized intensities during H_2_ oxidation by oxygen species at β-MnO_2_ surfaces in the presence of O_2_ are presented in Fig. 3. The primary difference due to the presence of O_2_ is that the normalized intensity of *v*(OH) increases with temperature in the presence of O_2_ (Fig. 3d), but decreases in the absence of O_2_ (Fig. 2d). It is also noted that H_2_O MS signal (3.0E-09) at 400 °C in the presence of O_2_ (Fig. 3c) is much stronger than that (1.48E-09) in Fig. 2c in the absence of O_2_. These differences in the normalized intensity of *v*(OH) trend and H_2_O MS signal are evidence of O_2_ involvement in H_2_ oxidation. O_2_ can promote not only the release of O in M^+^–O^2−^–M^+^ (Eq. (1)) but also the formation rate of M–OH from M^2+^–O^2**–**^ (Eq. (6)). With the increase in temperature, M–OH reacts with H_2_ (Eq. (7)) to form surface adsorption of H_2_O that desorbs into gaseous H_2_O at 250 °C (Eq. (8))^49^, resulting in the formation of terminal vacancies (bare Mn).

6 $$2{{{{{\rm{M}}}}}}{=}{{{{{\rm{O}}}}}}+{{{{{{\rm{H}}}}}}}_{2}\to 2{{{{{\rm{M}}}}}}{-}{{{{{\rm{OH}}}}}}$$

7 $$2{{{{{\rm{M}}}}}}{-}{{{{{\rm{OH}}}}}}+{{{{{{\rm{H}}}}}}}_{2}\to 2{{{{{\rm{M}}}}}}{-}{{{{{{\rm{OH}}}}}}}_{2}$$

8 $${{{{{\rm{M}}}}}}{-}{{{{{{\rm{OH}}}}}}}_{2}\to {{{{{\rm{M}}}}}}+{{{{{{\rm{H}}}}}}}_{2}{{{{{\rm{O}}}}}}$$

### Regeneration of H_2_-reduced β-MnO_2_ with Ar or O_2_

The fact that the normalized intensities of M^+^–O^2**–**^–M^+^ and M^2+^–O^2**–**^ are all negative during H_2_ oxidation in both H_2_/Ar and (H_2_ + O_2_) atmospheres (Figs. 2 and 3) indicates that OVs (M–□–M and M) can be generated even in the presence of O_2_. A similar result has been reported by Sun et al., where they found that M^+^–O^2**–**^–M^+^ and M^2+^–O^2**–**^ can be reduced by CO on ZnO^50^. The generation of the M–□–M and M may be due to either the decomposition rate of O_2_ at β-MnO_2_ surfaces is less than that of H_2_ oxidation or M–□–M and M cannot be regenerated. This issue was evaluated by conducting successive regeneration experiments in an Ar or O_2_/Ar atmosphere (Supplementary Table S2). β-MnO_2_ was first reduced by H_2_ in the TPR cell at 200 °C for 10 min, the regeneration was then carried out in an Ar or O_2_/Ar atmosphere by elevating the temperature from 25 °C to 400 °C.

**Fig. 4:**
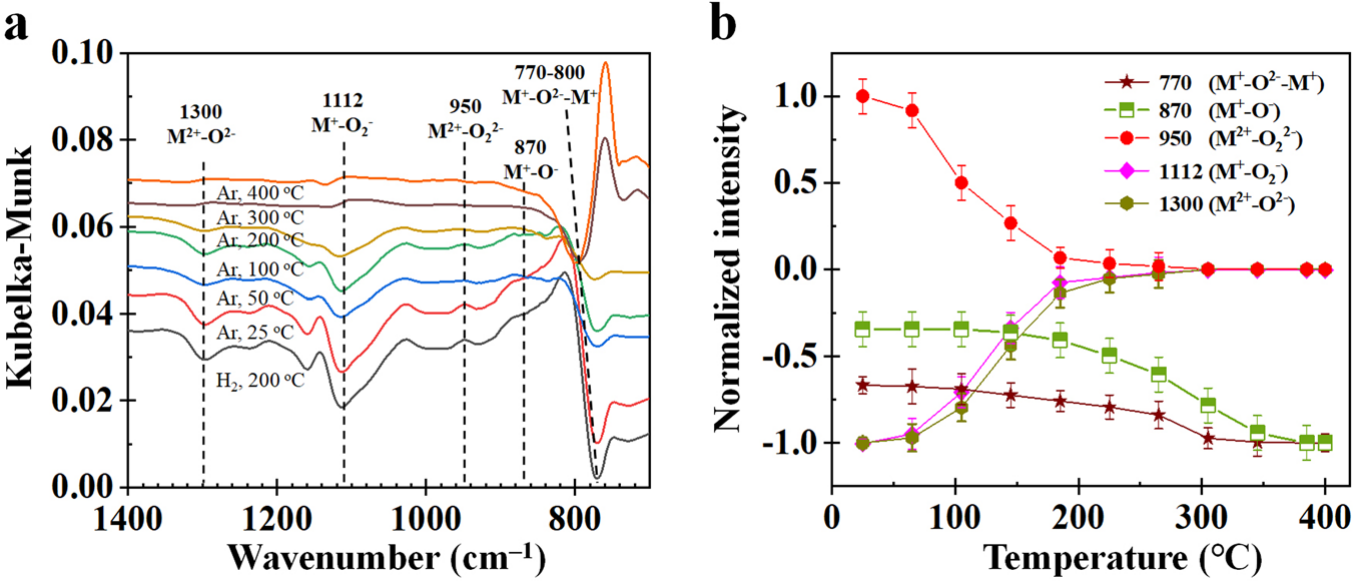
Experimental results of the regeneration of H_2_-reduced β-MnO_2_ in Ar at various temperatures. **a** DRIFTS spectra. **b** normalized intensities where the error bars are the standard deviations obtained by measuring the peak heights more than three times.

DRIFTS spectra and normalized intensities at various temperatures during the regeneration of H_2_-reduced β-MnO_2_ in the Ar atmosphere are presented in Fig. 4. When increasing temperature from 25 °C to 300 °C, the normalized intensity of M^2+^–O_2_^2−^ decreases rapidly to zero, while those of M^2+^–O^2**–**^ and M^+^–O_2_^−^ increase rapidly to zero (Fig. 4b). The normalized intensities of M^+^–O^2**–**^–M^+^ and M^+^–O^−^ asymptotically approach to –1.0 at a temperature close to 400 °C. This finding indicated that O atoms in M^+^–O^2**–**^–M^+^ and O_2_ molecules in M^2+^–O_2_^2−^ can migrate on β-MnO_2_ surfaces (Eqs. (9) and (10))^32^.

9 $${{{{{\rm{M}}}}}}{-}{{{{{\rm{O}}}}}}-{{{{{\rm{M}}}}}}+{{{{{\rm{M}}}}}}\to {{{{{\rm{M}}}}}}{-}{{\square }}{-}{{{{{\rm{M}}}}}}+{{{{{\rm{M}}}}}}{=}{{{{{\rm{O}}}}}}$$

10 $$\left({{{{{{\rm{O}}}}}}}_{2}^{2-}\right){{{{{\rm{M}}}}}} {-}{{\square }}{-}{{{{{\rm{M}}}}}}+{{{{{\rm{M}}}}}}{-}{{{{{\rm{O}}}}}}-{{{{{\rm{M}}}}}}\to {{{{{\rm{M}}}}}}{-}{{\square }}{-}{{{{{\rm{M}}}}}}\\ +\left({{{{{{\rm{O}}}}}}}_{2}^{-}\right){{{{{\rm{M}}}}}}{-}{{{{{\rm{O}}}}}}-{{{{{\rm{M}}}}}}$$

**Fig. 5:**
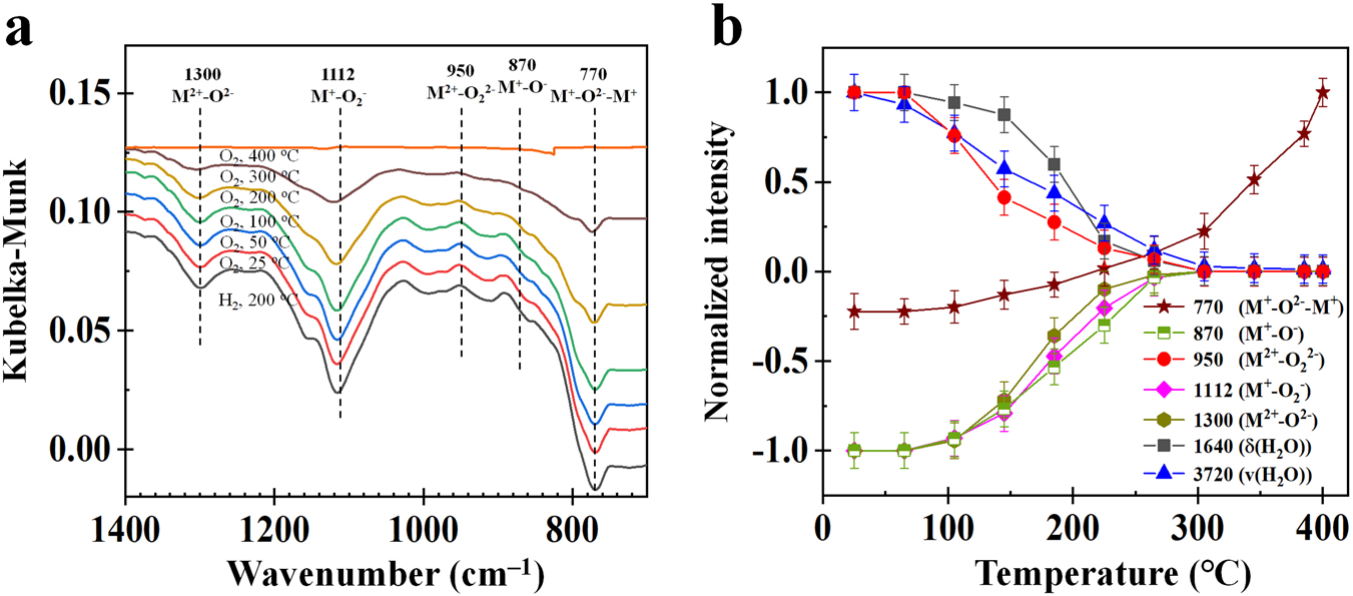
Experimental results of the regeneration of H_2_-reduced β-MnO_2_ in O_2_/Ar at various temperatures. **a** DRIFTS spectra. **b** normalized intensities where the error bars are the standard deviations obtained by measuring the peak heights more than three times.

DRIFTS spectra and normalized intensities at various temperatures during the regeneration of H_2_-reduced β-MnO_2_ in the O_2_/Ar atmosphere are illustrated in Fig. 5. The normalized intensity of M^+^–O^2**–**^–M^+^ increases from at a temperature higher than 100 °C, indicating that the regeneration of M^+^–O^2**–**^–M^+^ from M–□–M and O_2_ requires a temperature higher than 100 °C^51,52^. Furthermore, the normalized intensity of M^+^–O^2**–**^–M^+^ becomes positive at temperatures greater than 250 °C, at which all other surface oxygen species increase or decrease to 0.0, suggesting all other surface oxygen species have been completely regenerated. We may further note that M^+^–O^2**–**^–M^+^ can convert to M^2+^–O^2**–**^ (Eq. (9)). From the fact that the normalized intensity of M^2+^–O^2**–**^ increases little at temperatures greater than 250 °C but that of M^+^–O^2**–**^–M^+^ increases significantly, these findings deduce that the reaction in Eq. (9) is reversible.

The decreases in normalized intensities of *δ*(H_2_O) and *v*(OH) indicate that H_2_O can desorb from M–OH_2_ and M–OH at β-MnO_2_ surface, resulting in the formation of bare M.

### Roles of OVs in H_2_ catalytic oxidation

**Fig. 6:**
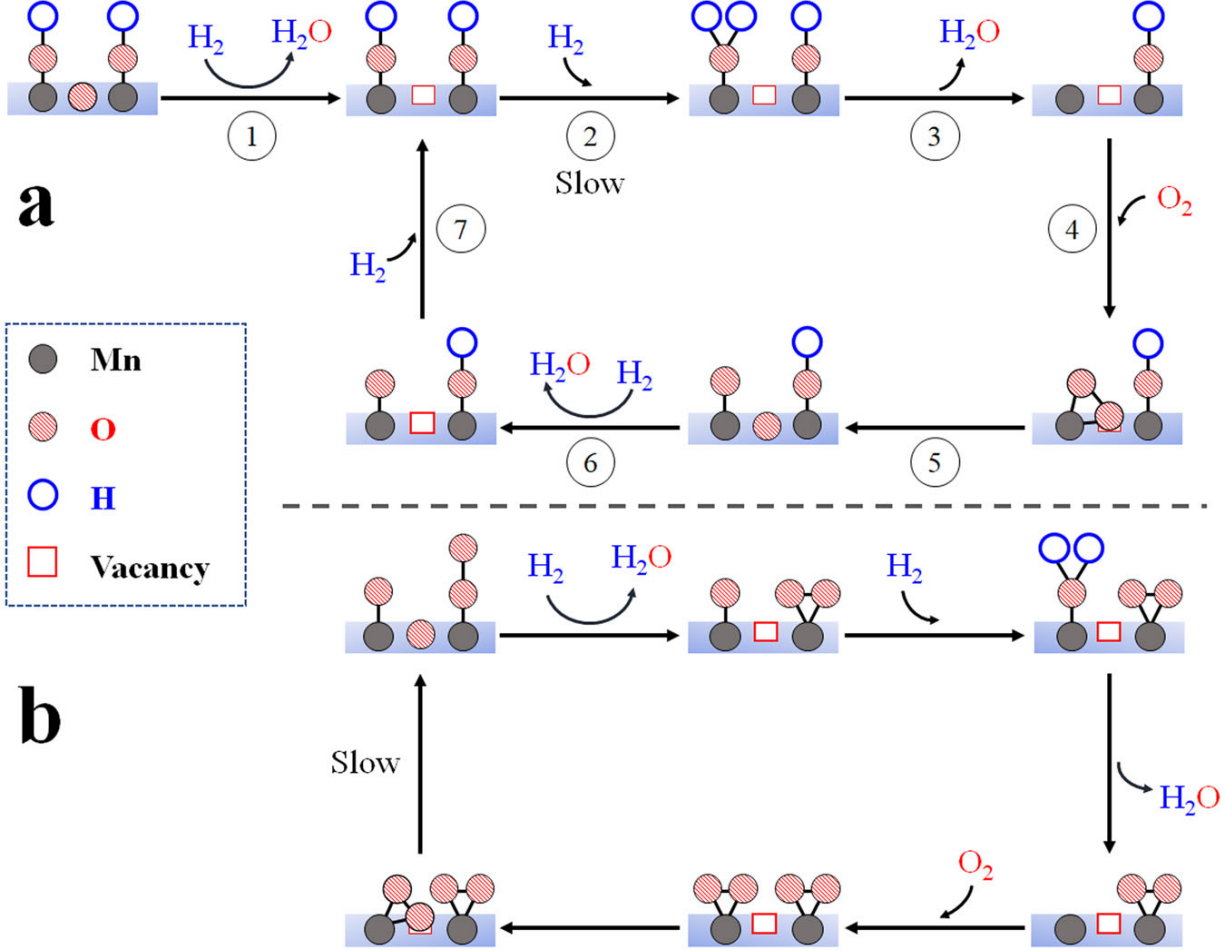
Roles and mechanisms of surface oxygen species and OVs in H_2_ oxidation at β-MnO_2_ surfaces. **a** H_2_ oxidation between 110 and 150 °C**. b** H_2_ oxidation at a temperature higher than 150 °C.

The roles of OVs in H_2_ oxidation process at β-MnO_2_ surfaces in the presence of O_2_ can be deduced from the above discussion, and the proposed mechanism is illustrated in Fig. 6. First, when the reaction temperature is in a range of 110–150 °C, the oxygen atom in the bridge-type M^+^–O^2–^–M^+^ can react with H_2_ to form H_2_O and OV via steps (1) and (6) in Fig. 6a. According to steps (2) and (7), the oxygen atoms in the terminal-type M^2+^–O^2–^ and M–OH react with H_2_ to generate surface M–OH and gaseous H_2_O. The gaseous O_2_ adsorbed at the bare M site in M^+^–□–M^+^ (step (4)) yields M^2+^–O_2_^2−^. M^2+^–O_2_^2−^ dissociates simultaneously to M^+^–O^2–^–M^+^ and M^2+^–O^2–^ (step (5), Eq. (11)). We can only find the decrease in M^+^–O^2–^–M^+^ and increase in M–□–M in this temperature range as the step (2) is rate limited reaction.

11 $$\left({{{{{{\rm{O}}}}}}}_{2}^{2-}\right){{{{{\rm{M}}}}}}{-}{{\square }}{-}{{{{{\rm{M}}}}}}\to {{{{{\rm{M}}}}}}{-}{{{{{\rm{O}}}}}}{-}{{{{{\rm{M}}}}}}{=}{{{{{\rm{O}}}}}}$$

As the oxidation process in Fig. 6b, when the temperature is above 150 °C, OH in M–OH becomes reactive enough, the O_2_ dissociation step (6) is slowest, resulting in the accumulation of M–□–M and M^2+^–O_2_^2−^.

### Conclusion

We explored O_2_ dissociation, OVs formation, and surface oxygen species conversion during H_2_ oxidation at β-MnO_2_ surface using the operando TPR-DRIFTS-MS technology. The results demonstrate that the operando TPR-DRIFTS-MS technology employed herein is a highly useful tool for identifying OVs at β-MnO_2_ surfaces and CeO_2_ and Co_3_O_4_ surfaces (Supplementary Fig. S6), and for understanding the roles of OVs and oxygen species in catalytic processes. In particular, the difference in the reaction characteristics of bridge-type (M^+^–O^2**–**^–M^+^) and terminal-type (M^2+^–O^2**–**^) oxygen species can be clearly observed using the operando TPR-DRIFTS-MS technology. Accordingly, we expect this technology could provide an important characterization method to understand the roles of surface oxygen species on metal oxide catalysts and enable the rational design of catalysts of OVs with satisfied performance.

## Methods

### Materials

β-MnO_2_ (99%) was purchased from Aladdin, Shanghai, China. Pure Ar (99.999%), pure O_2_ (99.999%), 5 vol% H_2_ standard gas (Ar balanced), and 5 vol% CO standard gas (Ar balanced) were purchased from Huayang, Changzhou, China.

### Catalyst characterization

Physicochemical properties of β-MnO_2_ were characterized by via various techniques, such as X-ray powder diffraction (XRD), thermogravimetric (TG) analysis, Raman, X-ray photoelectron spectroscopy (XPS), oxygen temperature-programmed desorption (O_2_-TPD), and high-resolution transmission electron microscopy (HRTEM).

### DRIFTS-MS system

A schematic diagram of the operando TPR-DRIFTS-MS system is shown in Supplementary Fig. S7. The system consisted of gas cylinders, gas flow meters (MFC, D07, Sevenstars Beijing, China), operando DRIFTS (Nicolet 50, Thermo Scientific, USA), and MS (Tilon LC-D200M, Ametek, USA). The DRIFTS was equipped with a TPR cell (HVC-DRP-5, Harrick, USA) and a narrow-band mercury cadmium telluride (MCT-A) detector with liquid nitrogen cooling for high sensitivity (0.09 cm^−1^) in collecting DRIFTS spectra between 4000 and 650 cm^−1^.

For the DRIFTS spectrum collection experiment, β-MnO_2_ powders were pretreated for 1 h in Ar at 450 °C (20 mL/min). Then, the β-MnO_2_ powders were cooled to room temperature and stabilized for 10 min, and DRIFTS background spectra were collected. The gas mixture (Supplementary Table S2) was supplied into the TPR cell for 20 min. The β-MnO_2_ powder temperature was elevated with programmed heating using a temperature controller. Series software was used to collect the corresponding spectra. Thirty-two scans were performed with a resolution of 4 cm^−1^, and the spectrum data of DRIFTS were analyzed using OMNIC software during the acquisition. The Kubelka–Munk function was used to convert the obtained spectra into absorption spectra, whose intensities were linearly related to the amount of adsorption. The gas from the TPR cell was analyzed using mass spectrometer (MS) (Tilon, LC-D200M, Ametek, USA) to obtain signals of H_2_ (*m*/*z* = 2), H_2_O (*m*/*z* = 18), O_2_ (*m*/*z* = 32), and Ar (*m*/*z* = 40).

### Normalization of peak intensity

The collected infrared spectra at different temperatures were normalized for relatively quantitative analysis. The normalization was calculated using Eqs. (12) and (13) based on the absolute values of the highest height (*P*_*i*_ _*max*_) for positive peaks and lowest peak height (*P*_*i*_ _*min*_) for negative peaks, respectively.

12 $${N}_{i}=\frac{{P}_{i}}{{P}_{i\,{max }}}$$

13 $${N}_{i}=\frac{{P}_{i}}{\left|{P}_{i\,{min }}\right|}$$

*N*_*i*_ represents the normalized intensity of the absorption peak *i* at the corresponding temperature; *P*_*i*_ represents the peak height of the absorption peak *i* at the corresponding temperature.

## Supplementary information

Supplementary Information

## Data Availability

Data will be made available on request.
