# Supplementary material for: Exploring the roles of oxygen species in H2 oxidation at β-MnO2 surfaces using operando DRIFTS-MS
Source: Commun Chem. 2022 Aug 20;5:97. doi: 10.1038/s42004-022-00717-0 (PMC9814464; doi:10.1038/s42004-022-00717-0)
Supplement: Supplementary file 1 — Supplementary Information [file 42004_2022_717_MOESM1_ESM.pdf]

Supplementary Materials for

Exploring the roles of oxygen species in H_2 oxidation at β-MnO_2 surfaces using operando DRIFTS-MS

Jiacheng Xu[1,2], Tiantian Zhang[1], Shiyu Fang[1], Jing Li[1,3], Zuliang Wu[1,3], Wei Wang[1,3], Jiali Zhu[1,3], Erhao Gao[1,3], Shuiliang Yao[1,2,3,*]

[1]School of Environmental and Safety Engineering, Changzhou University, Changzhou, China

[2]School of Material Science and Engineering, Changzhou University, Changzhou, China

[3]Advanced Plasma Catalysis Engineering Laboratory for China Petrochemical Industry, Changzhou, China

* Corresponding author. Email: yaos@cczu.edu.cn

Figs. S1 to S7

Tables S1 to S3

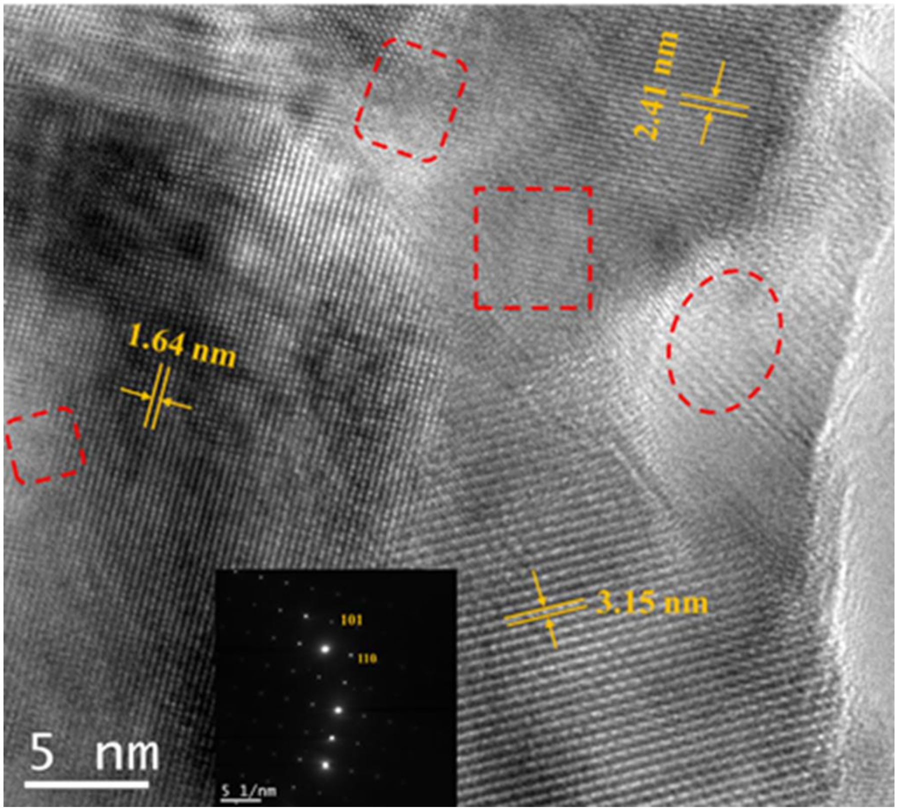

Fig. S1 HRTEM image of β-MnO_2.

In order to confirm the position of M=O at β-MnO_2, we have carried out the experiments of O_3 adsorption and decomposition on β-MnO_2. We found a peak at 1300 cm^{-1} becomes more and more obvious when increasing O_3 concertation (Fig. S2), indicating that M=O at 1300 cm^{-1} does exist at the surface of β-MnO_2.

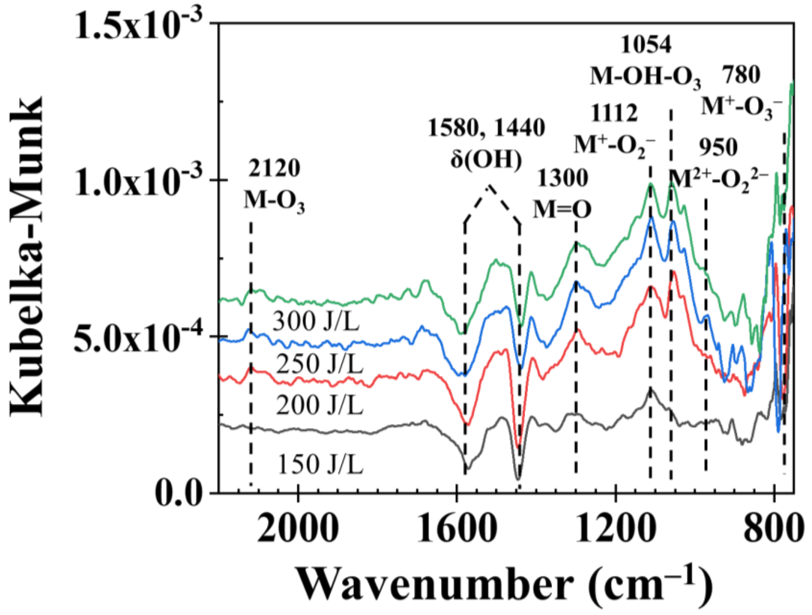

Fig. S2 DRIFT spectra of oxygen groups at β-MnO_2 at surfaces at different energy densities. Experimental conditions: 20vol.%O_2 and 80vol.%Ar, room temperature, O_3 concentration was changed by changing energy density (joules of electric energy per litter gas, J/L).

The DRIFTS spectra at various times under a fixed discharge condition or without discharges are shown in Fig. S3. As the discharge time increases, the peak height of M=O at 1300 cm^{-1} gradually increases and finally stabilizes. When the discharge is stopped, the peak height of M=O is basically unchanged, indicating that M=O is a strong adsorbed oxygen species (chemical adsorption).

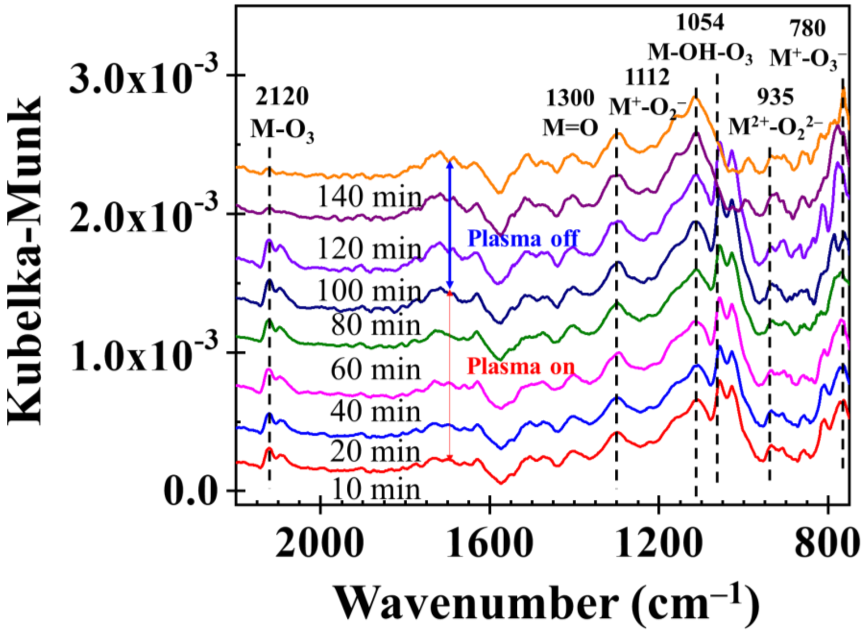

Fig. S3 DRIFT spectra of β-MnO_2 at different times with or without discharges (plasma on or off). Experimental conditions: 20vol.%O_2 and 80vol.%Ar, room temperature.

In order to investigate the reactivity of M=O, O_3 gas mixed with H_2 gas was fed to β-MnO_2 at various temperatures. When the temperature increases, the peak height of M=O gradually decreases. When the temperature reaches 150 °C, the peak height of M = O is negative (Fig. S4). This result obviously shows that the M=O from O_3 decomposition can react with H_2.

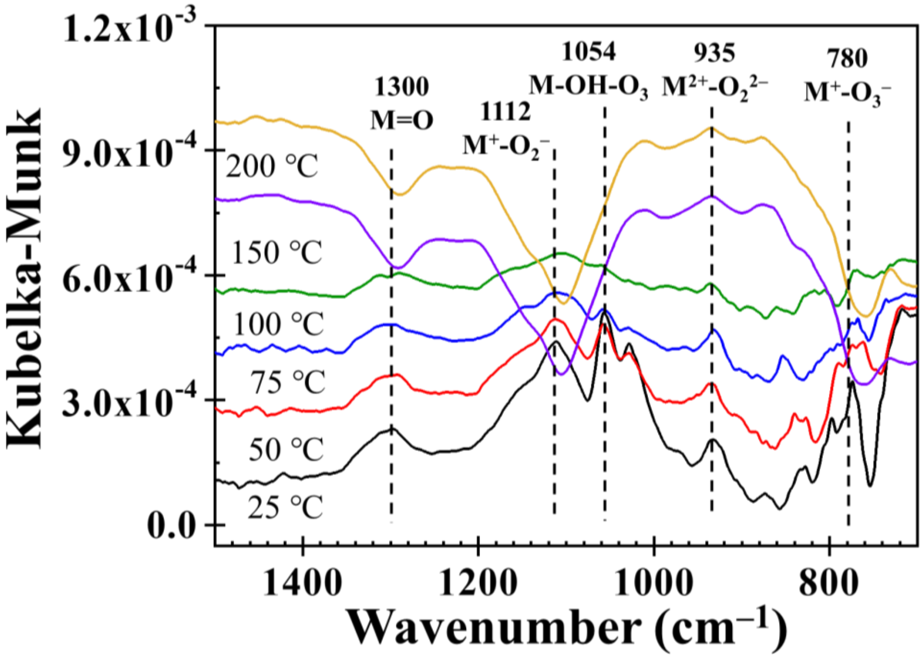

Fig. S4 DRIFT spectra of β-MnO_2 at different temperatures and a fixed O_3 concentration. Experimental conditions: H_2: 2.37%, O_2:10%, Ar: balance; temperature: 25-200 °C.

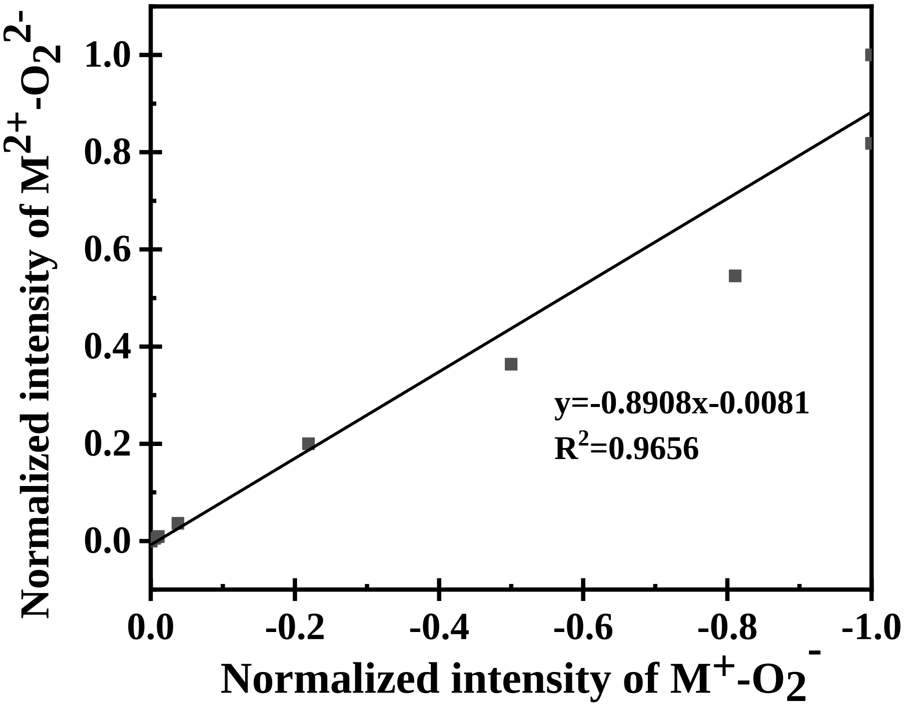

Fig. S5 Relation of normalized intensities of M^{2+}-O_2^{2-} and M^+-O^{2-}.

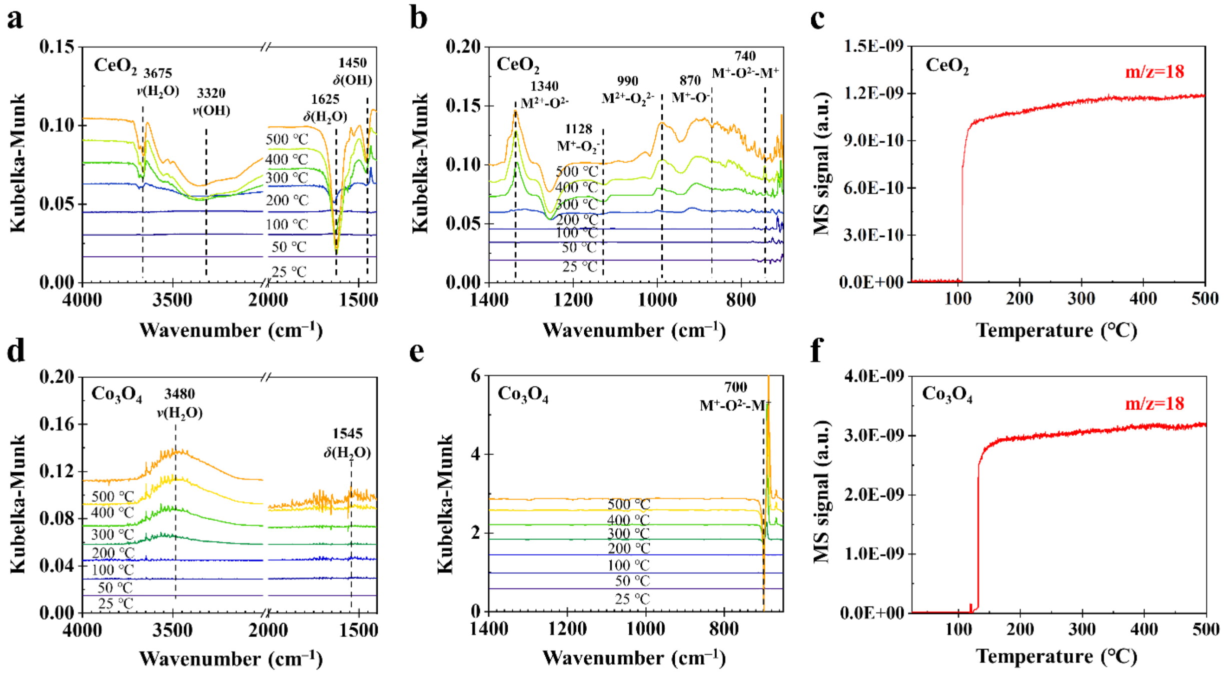

Fig. S6 Operando DRIFT-MS profiles of metal oxides (a-c CeO_2 and d-f Co_3O_4).

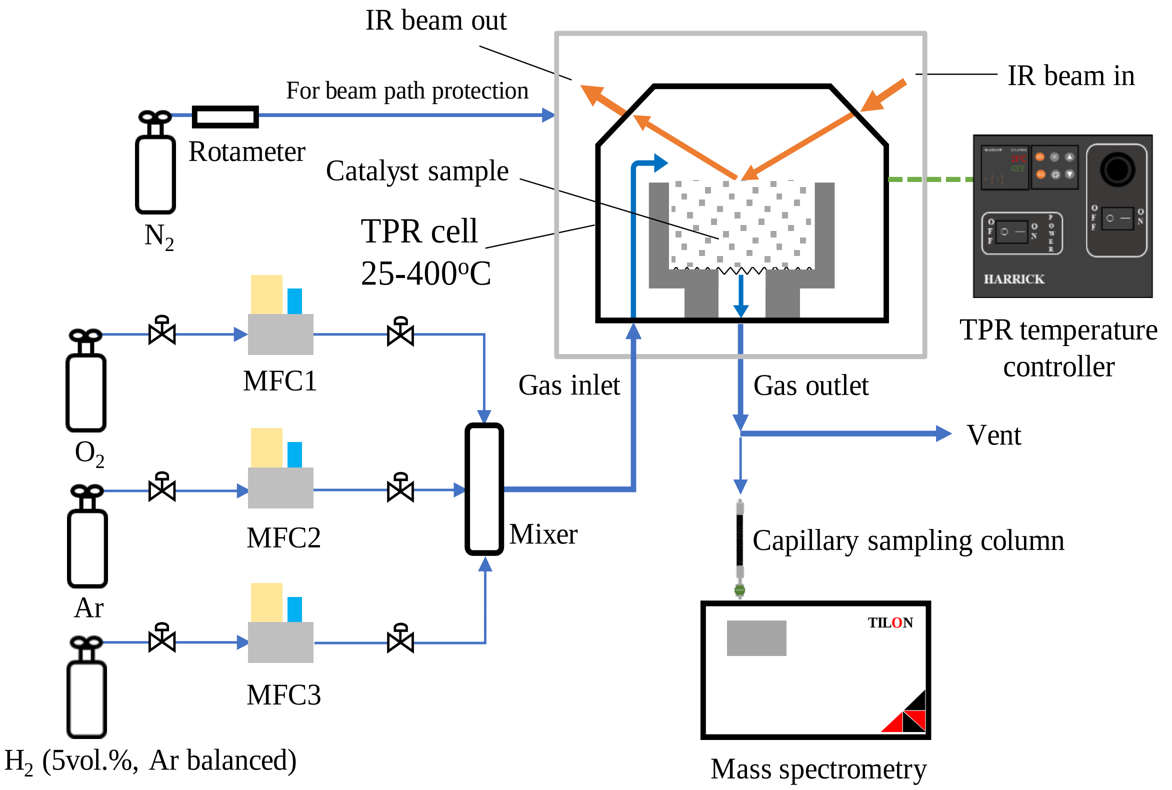

Fig. S7 Experimental setup of operando TPR-FTIR-MS system.

Table S1 Advantages and disadvantages of analytical methods for oxygen species and oxygen vacancies [1,2].

Method	Advantage	Disadvantage	Detection capacity
IR, Raman	Distinguish between different types of oxygen species and semi-quantitative	Short-lived species are difficult to measure	Different lattice configurations are distinguishable; surface species can also be differentiated
O_2-TPD/H_2-TPR	Distinguish between surface and bulk species, semi-quantitative	Cannot distinguish between different surface species	Distinguishes surface species from bulk
EPR	Substances containing unpaired electrons	Can only capture paramagnetic species	O_2^{2-}, $O_2^{\ -}$, O^{-}, and O_3^{-} are visible and distinguishable
DFT	Not limited by experiment and detectable performance	Need to simplify invalid assumptions	All oxygen species are distinguishable from each other
MS	Can measure the quantity of exchangeable oxygen and quantitative	Cannot distinguish between different surface species	Distinguishes surface species from bulk
X-ray Techniques	Surface and bulk species can be distinguished, semi-quantitative	Distinction between different oxygen species is difficult	Distinguishes surface species from bulk
Oxygen isotope	Determine which structure results in the conversion between easily exchangeable oxygen atoms and oxygen species	Different charged oxygen species cannot be distinguished	Can identify different types of surface species and lattice oxygen
TGA	Can indirectly judge and characterize oxygen vacancies in the crystal lattice, semi-quantitative	Cannot distinguish the type of reactive oxygen species	Indirect study of oxygen vacancies in the catalyst lattice
STEM	Can accurately qualitative oxygen vacancy	Cannot distinguish the type of reactive oxygen species and quantify	Determine the presence of oxygen vacancies
PALS	Sensitive to atomic-scale defects (vacancies, dislocations)	Cannot distinguish the type of reactive oxygen species and quantify	Obtain the internal microstructure and defect structure of the condensed matter

Table S2 Composition of the gases fed to the TPR cell.

Gas atmosphere	Composition (%)	Total gas flow rate (mL/min)
H_2/Ar	H_2: 2.37%, Ar: balance	38
H_2+O_2	H_2: 2.37%, O_2:10%, Ar: balance	38
O_2/Ar	O_2: 5.26%, Ar: balance	38
Ar	Ar:100%	20

Table S3 DRIFTS peaks and related functional groups.

Wavenumber (cm^{-1})	Functional group	Reference
750-800	M^{+}-O^{2-}-M^{+}, M-O-M	3
1300-1400	M^{2+}-O^{2-}, M=O	4-6
870	M^{+}-O^{-}, M-O	7
1110-1120	M^{+}-O_2^{-}	8, 9
930-960	M^{2+}-O_2^{2-}	10
1520, 1610, 1640	$\delta(H_2O)$, M-OH_2	11
3080, 3230, 3530, 3720	$\nu(OH)$	11, 12

Supplementary references

1. Ye, K., et al. An overview of advanced methods for the characterization of oxygen vacancies in materials. *Trend. Anal. Chem.* **116**, 102–108 (2019).
2. Williams, O. & Sievers, C. Active oxygen species in heterogeneously catalyzed oxidation reactions. *Appl. Catal. A-Gen.* **614**, 118057 (2021).
3. Panov, G., Dubkov, K. & Starokon, E. Active oxygen in selective oxidation catalysis. *Catal. Today* **117**, 148–155 (2006).
4. Wu, J., et al. In situ DRIFTS study of O_3 adsorption on CaO, γ-Al_2O_3, CuO, α-Fe_2O_3 and ZnO at room temperature for the catalytic ozonation of cinnamaldehyde. *Appl. Surf. Sci.* **412**, 290–305 (2017).
5. Brodu, N., et al. Role of Lewis acid sites of ZSM-5 zeolite on gaseous ozone abatement. *Chem. Eng. J.* **231**, 281–286 (2013).
6. Roscoe, J. & Abbatt, J. Diffuse Reflectance FTIR Study of the Interaction of Alumina Surfaces with Ozone and Water Vapor. *J. Phys. Chem.* A. **109**, 9028–9034 (2005).
7. Larson, V., et al. Iron and manganese oxo complexes, oxo wall and beyond. *Nature Rev. Chem.* **4**, 404 (2020).
8. Li. C., Domen. K., Maruya. K. Oxygen exchange reactions over cerium oxide: An FT-IR study. *J. Catal.* **123**, 436–442 (1990).
9. Jiang. S., et al. Insight into the reaction mechanism of CO_2 activation for CH_4 reforming over NiO-MgO: A combination of DRIFTS and DFT study. *Appl. Surf. Sci.* **416**, 59–68 (2017).
10. Luca. B., Alfonso. P., Maria. C. O_2 activation over Ag-decorated CeO_2(111) and TiO_2(110) surfaces: a theoretical comparative investigation. *J. Phys. Chem. C.* **124**, 25917–25930 (2020).

11. TheoKloprogge. J., et al. XPS study of the major minerals in bauxite: Gibbsite, bayerite and (pseudo-) boehmite. *J. Colloid. Interf. Sci.* **296(2)**, 572–576 (2006).
12. Gonzalez. P. & Calatayud. M. Toward an understanding of the hydrogenation reaction of MO_2 gas-phase clusters (M = Ti, Zr, and Hf). *J. Phys. Chem. C.* **117 (25)**, 5354–5364 (2013).
